# Analysis of combinatory effects of free weight resistance training and a high-protein diet on body composition and strength capacity in postmenopausal women - A 12-week randomized controlled trial

**DOI:** 10.1016/j.jnha.2024.100349

**Published:** 2024-09-03

**Authors:** Paulina Ioannidou, Zsuzsanna Dóró, Jan Schalla, Wim Wätjen, Patrick Diel, Eduard Isenmann

**Affiliations:** a Institute for Cardiovascular Research and Sports Medicine, Department of Molecular and Cellular Sports Medicine, German Sports University Cologne, Cologne, Germany; b Department of Fitness and Health, IST University of Applied Sciences, Dusseldorf, Germany; c Biofunctionality of Secondary Plant Compounds, Institute of Agricultural and Nutritional Martin-Luther-Universität Halle-Wittenberg, Halle, Germany

**Keywords:** Ageing, Menopause, Strength, Resistance training, Muscle mass, Protein supplementation

## Abstract

**Background:**

Menopause has a significant impact on the endocrine system of middle-aged women, resulting in a loss of skeletal muscle mass (SMM), changes in fat mass (FM) and a reduction in strength capacity. Resistance training (RT) and a high-protein diet (HPD) are effective methods for maintaining or increasing SMM. This study aims to determine the effects of HPD and RT on body composition, muscle thickness and strength capacity in postmenopausal women.

**Methods:**

In total 55 healthy postmenopausal women (age: 58.2 ± 5.6 years, weight 69.1 ± 9.6 kg, height 166.5 ± 6.5 cm) successfully participated in the study. The women were randomly assigned to either group: training + protein (2.5 g/kg fat-free mass (FFM)) (n = 15; TP); only training (n = 12; T); only protein (2.5 g/kg FFM) (n = 14; CP) or control (n = 14; C). TP and T performed RT for 12 weeks with three training sessions and five exercises each. CP and C were prohibited from training during the period. The main parameters analysed for body composition were FFM, SMM, FM, muscle thickness of the M. rectus femoris, M. biceps femoris, M. triceps brachii and M. biceps brachii muscles. Strength was tested using a dynamometer for grip strength and 1-RM in the squat (BBS) and deadlift (DL).

**Results:**

The SMM significantly increased by RT (TP: (Δ+1.4 ± 0.9 kg; p < 0.05; d = 0.4; T: Δ+1.2 ± 1.3kg; p < 0.05; d = 0.3) and FM could be reduced only in T: (Δ−2.4 ± 2.9 kg; p < 0.05; d = 0.3). In muscle thickness a significant increase in the M. biceps brachii in both training groups (TP: (Δ+0.4 ± 0.3 cm; p < 0.05; d = 1.6; T: (Δ+0.3 ± 0.3 cm; p < 0.05; *d =* 0.9) and in M. biceps femoris only in TP (Δ+0.3 ± 0.4 cm; p < 0.05; *d =* 0.9) were observed. HPD without training does not affect body composition, A significant increase in grip strength (TP: Δ+4.7 ± 2.4 kg; (p < 0.05; *d* = 1.5; T: (Δ+3.6 ± 3.0 kg; p < 0.05; *d* = 0.8), in BBS (TP: (Δ+30.0 ± 14.2 kg; p < 0.05; *d* = 1.5; T: (Δ+34.0 ± 12.0 kg; p < 0.05; *d* = 2.4) and in DL (TP: (Δ+20.8 ± 10.3 kg; p < 0.05; *d* = 1.6; T: (Δ+22.1 ± 7.6 kg; p < 0.05; *d* = 2.0) was observed in both training groups. The CP also recorded a significant increase in the BBS (Δ+7.5 ± 5.4 kg; p < 0.05; *d* = 0.4) and in DL (Δ+5.5 ± 7.7 kg; p < 0.05; *d* = 0.5). No significant differences were detected for TP and T for any of the parameters.

**Conclusion:**

The results indicate that RT enhances body composition and strength capacity in postmenopausal women and is a preventive strategy against muscle atrophy. Besides HPD without training has a trivial significant effect on BBS and DL. HPD with RT has no clear additive effect on body composition and strength capacity. Further studies are needed to confirm these observations.

## 1 Introduction

Life expectancy has been steadily increasing for decades and it is known that physical performance decreases and body composition changes in the fourth decade of life [[1], [2], [3], [4]]. Sexual hormones play an important role as well in women and in men in this context. Especially, sex hormones such as luteinizing hormone, follicle-stimulating hormone, estradiol (E2) and progesterone (P) dramatically change in the fifth to sixth decade of life resulting in menopause and the end of fertility in women [2,5]. The decrease in E2 concentration results in a greater decline in skeletal muscle mass (SMM) and bone mineral density (BMD) [2]. Due to the reduction in SMM, maximum strength and functionality are negatively affected and decrease significantly [3]. Consequently, postmenopausal women have a 2.1-fold higher risk of falling and a 2.7-fold higher risk of fractures than premenopausal women [4]. In addition, they have a higher risk of developing osteoporosis and other musculoskeletal disorders [5].

To maintain SMM and promote overall longevity, physical activity and especially resistance training (RT) is a key preventative strategy against the ageing process [6]. One aspect is the influence of RT on body composition, although this is not yet fully elucidated. While some studies suggest a small to moderate increase in lean body mass (LBM), there are no clear effects on fat mass (FM) [7]. These findings are in line with observations of Sa et al., which indicate that a reduction in FM is more evident with combined RT and endurance training [8]. Besides, initial studies have shown that RT twice a week over 10 weeks is not sufficient to increase fat-free mass (FFM) or SMM in postmenopausal women but in premenopausal women [9]. In terms of strength, however, similar significant increases can be achieved in the lower and upper body in pre- and postmenopausal women [9].

Regarding, RT methodology only a few studies have been carried out on free weights [[7], [8], [9], [10], [11]]. Free weight exercises are close to functional and everyday movements and can effectively simulate applied scientific principles [12]. Besides, it has also been shown to be a safe and effective method of increasing strength in postmenopausal women [9].

In addition to strength training, protein intake to maintain or increase SMM is also the subject of lively debate. The combined effects of RT and protein intake have also been investigated in postmenopausal women. Many studies focused on additional protein intake on training days or examined the timing of protein supplementation during a strength training program [9,[13], [14], [15], [16], [17], [18]]. In general, no significant differences were observed between the RT and the RT with protein application in terms of strength capacity and body composition. Besides Rossato et al. detected no additive effects on strength capacity and body composition through a high protein diet (HPD) (1.2 g/kg BW per day) in a ten-week training study with three training sessions per week [16]. Studies with more than 1.2 g/kg BW and strength training have not yet been carried out with post-menopausal women. Potential side effects are often discussed concerning an HPD [18]. However, initial studies in young strength athletes show that a super HPD over one year has no negative effects on liver and kidney function [19]. On the other hand, it is also known that liver and kidney function decline with age [[20], [21], [22], [23]], so side effects could potentially also be caused by HPD.

Based on the existing research, a four-arm randomized trial was conducted on the effects of strength training with free weights and HPD on strength and body composition in postmenopausal women. In addition, exploratory data was collected on the effects of HPD on liver and kidney function to identify potential side effects.

## 2 Material and methods

### 2.1 Participants

To determine the sample size, a power analysis (F-tests, ANOVA Fixed effects, special, main effects, and inter-action) was performed a priori. For the calculation, a medium to strong effect (f) (0.25–0.40), an α-error of 0.05, and a power of 0.8 (1-β error) were specified based on previous studies with similar study design and parameters [9,24]. Due to the four-arm study design (df = 3), a total sample size of 24–48 subjects was calculated. However, since the study lasted for a total of 14 weeks (two weeks of pre- and post-testing and 12 weeks of intervention) and drop-outs were taken into account, the number of subjects was set at a minimum of 50.

In total a population of 63 postmenopausal women was recruited. Eight individuals (n = 8) dropped out due to lack of commitment (n = 3), illness (n = 2), or no more interest (n = 3). All participants (n = 55) completed health history questionnaires and signed a consent form to evaluate potential exclusion criteria. Inclusion criteria were: >50 years, last period over two years ago, physically independent, had no cardiac, orthopedic, or musculoskeletal dysfunction that could impede physical exercise; not having uncontrolled diabetes mellitus or hypertension; not receiving hormonal replacement therapy; and not be involved in the practice of regular physical activity performed more than once a week over the three months before the start of the study. The questionnaire included basal anthropometric data, use of drugs and medication, last episode of the menstrual cycle, and general medical conditions such as operations, orthopedic, cardiologic, or other medical conditions. The classification of the participants as postmenopausal was based on hormone concentrations referring to low E2 and low P, as well as their last menstruation beyond two years [25]. Measuring hormone concentration in saliva is an established method to verify a menstrual cycle [25,26]. This has also been used in previous studies of postmenopausal women [9].

In a two-week familiarization phase participants were introduced to track their energy intake via mobile phone application (Fatsecret Secret Industries Pty Ltd, Caulfield North, Victoria, Australia) and the movements of the exercises.

### 2.2 Randomization

Each participant was anonymized through an alphabetic and numeric code. After the recruitment, all codes were digitally set through a non-limited randomization (www.randomizer.org, 2022). All participants were cleared to their groups. Trainers and those who assessed outcomes were blinded to the groups.

### 2.3 Experimental design – study design

The study was approved by the local ethics committee of the German Sports University Cologne, Cologne, Germany (22/2022) according to the Declaration of Helsinki and registered in the German Registry of Clinical Studies (17/02/2022; DRKS00023826). Additionally, all measures and regulations were approved including the hygiene concept for the prevention of infection to COVID-19 by the local authorities.

The study design consisted of a four-arm model. The groups are divided into two training groups and two groups without training. The participants were randomly assigned to either the training group with a high-protein diet (TP), the training group without dietary requirements (T), the control group with a high-protein diet (CP) or the control group without dietary requirements (C).

At the beginning (T0) of the study and after 12 weeks (T1), body composition, muscle thickness and static and dynamic maximum strength in the lower body were measured in a single-blinded study. During the initial measurement, a saliva sample was also taken from all participants and a blood sample from the CP group only. All tests were carried out in the morning, rested and fasting between 7.00 and 10.00 am. However, the participants had to drink 300−500 ml of water after getting up in the morning to compensate for their water loss overnight. The test days proceeded as follows:

 Station A: Reception, explanation of study objective, design and framework (only on T0)

 Station B: Collection of saliva sample (only at T0), blood sample (only CP)

 Station C: Measurement of body weight and body composition

 Station D: Muscle thickness measurement via ultrasound analysis

 Station E: Static grip strength via dynamometer

 Station F: Dynamic maximum strength box back squat (BBS)

 Station G: Dynamic maximum strength deadlift (DL).

**Fig. 1:**
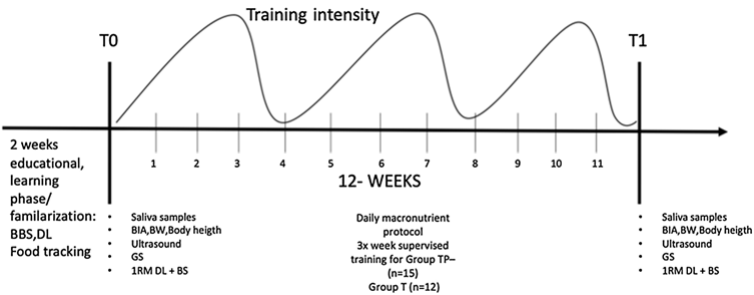
Schematic representation of study design. BBS = box Back Squat; DL = Deadlift, BIA = bioelectrical impedance analysis; BW: bodyweight; GS = Grip strength; 1RM = One repetition maximum; TP = Training + Protein group, T = Training group.

After T0, supervised RT with TP and T was performed. The control groups CP and C were not allowed to perform RT during the intervention period. All groups tracked their diet over the entire period using a mobile phone app (Fig. 1).

### 2.4 Bodyweight and body composition

The bodyweight was measured via Seca body scale (kg) (Seca 813 – Seca Deutschland 22089 Hamburg) and the height with a Seca wall measuring tape. The bodyweight and height are measured in underwear. Body composition was determined via bio-impedance analysis (BIA) (Juwell 600 – Body Explorer Premium Health Conceps Firma Siegfried Molnár A-7000 Eisenstadt). The body composition included the measuring of the total body water (TBW), FFM, SMM and FM [27,28].

### 2.5 Muscle thickness

After analyzing the body composition, ultrasound measurement for muscle thickness was conducted. To determine a visual value of muscle thickness, measurements were performed with a B-mode ultrasound (Logiq C5 GE Healthcare Chicago, Illinois, USA) and a 10 MHz linear probe (8L H4001DB Transducer GE Healthcare Chicago, Illinois, USA) [29].

The standardization for four measuring points, M. rectus femoris (RF), M. biceps femoris (BF), M. triceps brachii (TB) and M. biceps brachii (BB) was dimensioned by 50% of the following stretch by using a waterproof marker: The RF and BB were measured with the participants lying supine. For the RF, palpating the spina iliaca anterior inferior and proximal border of the patella and taking 50% of the distance (gain 50db; image depth 4.5 cm). The BB was measured by 50% of the length starting from the anterior acromion to the radius head (gain 50db; image depth 4.5 cm).

For measurements of the BF and TB, the participants lay in a prone position. The BF distance was measured by the distance of 50% of the caudal sciatic tuber to the lateral head of the fibula (gain 50 db; image depth 6 cm). The TB was measured at 50% from the laterodorsal part of the acromion to the proximal palpation of the olecranon (gain 50 db; image depth 4.5 cm). Following the Delphi-Based Consensus Statement [30] muscle measurements, we applied the ultrasound transmission gel to the transducer, pointing the transducer marker cranial, longitudinal on the extremity. The Probe did not apply pressure on the underlying tissue while maintaining a 90° angle to the muscle.

The images were recorded and reviewed by OsiriXLite (Pixmeo SARL, 266 Rue de Bernex, CH-1233 Bernex, Switzerland). The images pre- and post-intervention were performed by the same technician, who was blind to the group identity of each participant.

### 2.6 Grip strength – dynamometer

After ultrasound analyses, static grip strength was measured. A grip strength (GS) dynamometer was used for the static strength test. Using the given standardized protocol for GS hand-held dynamometer (digital Jamar+, Fabrication Enterprises, New York, United States) through previous studies [9]. The participants were seated upright on a chair, placing the elbow of the tested hand 90° bent in contact to the body. The instructions were clearly defined by pressing maximally for five seconds, three repetitions, and a rest period of 120 s for the right side. GS was performed before the lifts to avoid muscular fatigue of the hand flexors. The GS replaces the testing of the other exercises but can still provide valid information about a potential increase in GS in the respective groups.

### 2.7 Dynamic strength test

For dynamic strength, the one repetition maximum (1 RM) in BBS (50.8 cm) and DL were evaluated. Participants started with the BBS and a technical barbell was used to facilitate the back squat technique (Technique Barbell Aluminium-Steel SQMIZE® OB72ALS7.5, 7.5 KG – Simple Products GmbH, Seevetal). The standardized height for the squat was a 50.8 cm (20 inch) box for the 1 RM testing. During the 12-week training period, the participants trained with the same height of the box.

After the 1 RM BBS test, the 1 RM in DL was performed. For the warm-up and to verify the technical requirements, kettlebells from 6−20 kg (Rogue Fitness Europe - Pori) to the 20 kg Barbell (Eleiko XF Bar, Eleiko Sweden Stockholm) were used. Subsequently, a standardized barbell (20 kg, Eleiko XF Bar Sweden Stockholm) and weight plates (Eleiko XF bumper plate diameter is 450 mm, Eleiko Sweden Stockholm) were used for the maximum strength test.

The maximum strength test (1 RM) was performed modified after the NSCA guide to tests and assessments [31]. A 5-minute standardized warm-up included air squats and a DL technique with an empty barbell or kettlebell. The weight is progressively increased and raised to the 1RM. Here an individual increase is made, current values of the NSCA are not representative of an untrained population of postmenopausal women. A target increase of 5–10% is aimed to avoid potential overload [[31], [32], [33]].

### 2.8 Hormone analyses

Hormone concentrations of E2 and P were determined in saliva using the salivary estrogen and progesterone ELISA kits (Enzyme-linked Immunosorbent Assay, Ref: SLV 2931 and SLV 4188) [25]. Saliva samples were taken in the morning (7.00–10.00) on a fasting basis and then stored in a −20-degree freezer until analysis.

### 2.9 Blood analyses

Blood samples (9 ml) were taken from the CP at the beginning and end of the intervention to determine whether the HPD affected liver and kidney function. Alanine aminotransferase (ALT), Aspartate aminotransferase (AST) and Gamma glutamyl transferase (GGT) were analyzed for liver function and creatinine concentration for kidney function. The blood samples were analyzed by the external routine laboratory Dr. Wisplinghoff (Horbeller Str. 18 – 20 50858 Köln, Germany).

### 2.10 Intervention

The intervention phase was carried out over 12 weeks and included three training sessions per week [34]. The training sessions were conducted from Monday to Saturday with a break of 48−72 h between the training days. Due to the pandemic and the coronavirus restrictions, the training days and training groups were determined before the start of the intervention. All training sessions were supervised by a qualified personal trainer in a ratio of 1:2–4. Weight adjustment is done according to the Repetitions in Reserve (RIR) principle [35]. Beginning with an eight-kilo kettlebell all participants should evaluate their subjective rate of exertion and how many repetitions they could potentially target on a goblet squat variation. Using the 10 kg barbell from blocks to obtain the standardized height, an increase in the beginning from 20% to 5% on higher intensities were obtained. During the training sessions, the participants started with the BBS or DL depending on the available training station. The use of reps in reserve (RIR) was observed to be an easier tool for estimation on non-experienced athletes [36]. RIR focuses on the potential reserves, i.e. possible repetitions. RIR is defined by a numerous scale of potential possible repetitions remaining before reaching muscular failure (10 RIR implements 10 repetitions before muscle failure, a targeted 5 RIR defines a potential muscle failure on 5 repetitions) [36]. The goal of using RIR is to determine how close or far participants are from muscle failure, which indicates the level of effort in each set. Efficacy and safety of supervised training is well observed to engage maximum outcome during heavy lifts and safety in case of muscle failure [34,35,37,38].

### 2.11 Resistance training protocol and exercises

A detailed description of the RT protocol (sets, repetitions, intensity and rest) throughout the study can be found in the appendix.

To improve the strength capacity as well as prevent injuries, we implemented three more exercises besides the BBS and DL: The lat pull-down on a machine with a shoulder width grip, sitting upright and knee flexion around 90°, lateral dumbbell raises standing neutral and lifting arms up to 90°, single arm lateral walking carries. These exercises provide additional muscular capacity to improve the BBS and DL and injury prevention of the lower back [39]. The training schedule was planned over 12 weeks and divided into three blocks. Each block was divided into four weeks. The first two weeks implemented a slow progression where we aimed to set the last set with three repetitions with 1–2 RIR and a weekly progression of 5–10% on the last set. There was no focus on eccentric movement, the speed of the barbell was performed with a one-second concentric phase, one-second hold phase and one-second eccentric phase. This principle was also transferred to the lat pull-down and lateral raises.

Week three was scheduled as a peak weak, participants experienced lower volume and higher intensities. Peak week progressions were targeted to hit their 1 RM BBS and 1 RM DL or surpass their 1 RM weight. The fourth week implemented the deload week where weights and RIR were scaled down to 30% for a regenerative purpose. The supervisors were responsible for ensuring adherence to the set RIR and the corresponding weight adjustment and accomplished this by engaging with the participants and examining the execution of the exercises.

We implemented an undulating periodized program with weekly adaptions as described above to improve strength, hypertrophy on a short time and manage load and regeneration [40,41].

Each Participant started with BBS. The warm up included for every exercise the same scheme of ten repetitions, low load at 8–9 repetitions in reserve. Followed by the DL, the lat pulldown, lateral raises and unilateral carries. The only adaptation of the training scheme was the unilateral carries where the participants walked 15 m for each side for four sets. There was no specific speed requirement for eccentric or concentric movements. The pauses between sets varied from the intensity of the set: sets with a lower intensity of eight RIR implemented a pause of 60 s, and more intense sets with a target of 1–2 RIR resulted in a pause of 180 s [42,43].

A total of 36 training sessions were targeted. On the first week (training sessions 1–3, block one), the fifth week (training sessions 13–15, block five) and nine (training sessions 25–27, block nine) the BBS, DL, lat pulldown, and lateral raises were performed with six sets which included the warm-up sets. The unilateral carries were permanently completed with four sets of 15 m back and forth.

Set one was performed with ten repetitions, with eight RIR and a break of 60 s. Set two was performed with eight repetitions, with seven RIR and a break of 60 s. Set three was performed with six repetitions an estimated four RIR and a break of 90 s. Set four was performed with five repetitions and a targeted RIR of three and a break of 90 s. Set five and six included three repetitions and only 1–2 RIR and a break of 120–180 s dependently if the participants hit three repetitions at two RIR or failed to perform 3 repetitions.

The second block included training sessions 4, 5, 6, 16, 17, 18, 28, 29 and 30. This block is similar to block one, with a 5–10% target of increased weights. Block three increased the intensity of a peak week (training sessions 7, 8, 9, 19, 20, 21, 31, 32, 33). Six sets were performed including the warm-up sets 1–3 with a repetition target of 10, 6 and 6 repetitions and a decreasing RIR of 8, 7 and 4. The last sets four and five aimed an intensity of 1–2 RIR and three repetitions. The last sixth set was performed with two repetitions and 1 RIR. The last block (training sessions 10, 11, 12, 22, 23, 24, 34, 35, 36) aimed to mimic a deload week with decreased weights (−10% in sets 4–6) and increased RIR. Sets 1–3 were similarly performed as warm-up sets with 10, 8 and 6 repetitions and 8, 7 and 4 RIR. The last sets four five and six included 6, 5 and 5 sets with an RIR of 3.

### 2.12 Nutrition

All the participants monitored their daily energy intake and macronutrient distribution via a food tracking application (Fatsecret Secret Industries Pty Ltd, Caulfield North, Victoria, Australia) [44,45] over the entire period. TP and CP followed a high-protein diet. Due to different body compositions, the protein intake was calculated on the FFM [[45], [46], [47]]. The daily protein intake was set at least 2.5 g/kg FFM [48]. To ensure protein intake, foodstuffs such as protein-rich cheese (Quäse, Loose GmbH) and certified protein powder (GermanProt 9000 - Whey Protein Isolate, Sachsenmilch Leppersdorf GmbH, Germany) were available to the participants. The whey protein was used in previous studies and was checked for purity and quality before the intervention [9]. No specifications were given for the other macronutrients.

Additionally, after each training session, TP had to drink 40 g of maltodextrin and 30 g of the protein powder provided throughout the entire study to increase the resorption of Leucine [[48], [49], [50]]. CP added also whey isolate if they did not hit their protein target per day.

### 2.13 Statistical analysis

The statistical analysis was performed with R and R-Studio (Version: 4.3.0). All dependent variables were tested checked for normal distribution as well as variance homogeneity between groups by visual inspection of QQ-Plots and Boxplots respectively.

The Variables BW, FFM, SMM, FM, GS, BBS, DL, RF, BF, BB and TB were analyzed using linear mixed-effects (LME) models using the lme4 package [51]. Furthermore, the influence of a HPD on liver function and kidney function was examined in the CP group with a dependent *t*-test.

For all LME-models, the fixed effects Group (C, CP, T, TP), Time (T1, T2) and their respective interaction terms are included in each model. Random effects are specified to have a random intercept for each Subject. After Model fitting, using the emmeans Package [52], post-hoc tests for the corresponding model were calculated. Multiple comparison adjustments were done using the false discovery rate. In addition, a pairwise comparison of the changes (Δ) in the respective groups was carried out for each variable. The significance threshold is set to p < 0.05. In addition, the effect size for significant time effects was calculated according to Cohen's d [53,54].

The effect size was categorized according to Rhea's classification for untrained, recreationally trained and highly trained [55]. As all participants were not experienced in strength training at the beginning, the following classification for untrained was used: trivial: <0.5; small: 0.5 ≤ 1.25; moderate: 1.25 ≤ 2.0; high: >2.0.

## 3 Results

### 3.1 Baseline characteristics

The baseline characteristics (T0) of the participants in the individual groups are shown in Table 1.

**Table 1:** Baseline characteristics.

Group	TP (n = 15)	T (n = 12)	CP (n = 14)	C (n = 14)
Age (years)	57.0 ± 6.1	57.6 ± 5.1	56.8 ± 5.4	60.7 ± 4.8
Height (cm)	166.5 ± 6.6	166.6 ± 6.7	167.3 ± 6.7	165.1 ± 6.9
Bodyweight (kg)	71.6 ± 8.9	68.1 ± 7.6	65.3 ± 7.7	73.0 ± 11.4

### 3.2 Body composition

#### 3.2.1 Bodyweight (BW)

**Fig. 2:**
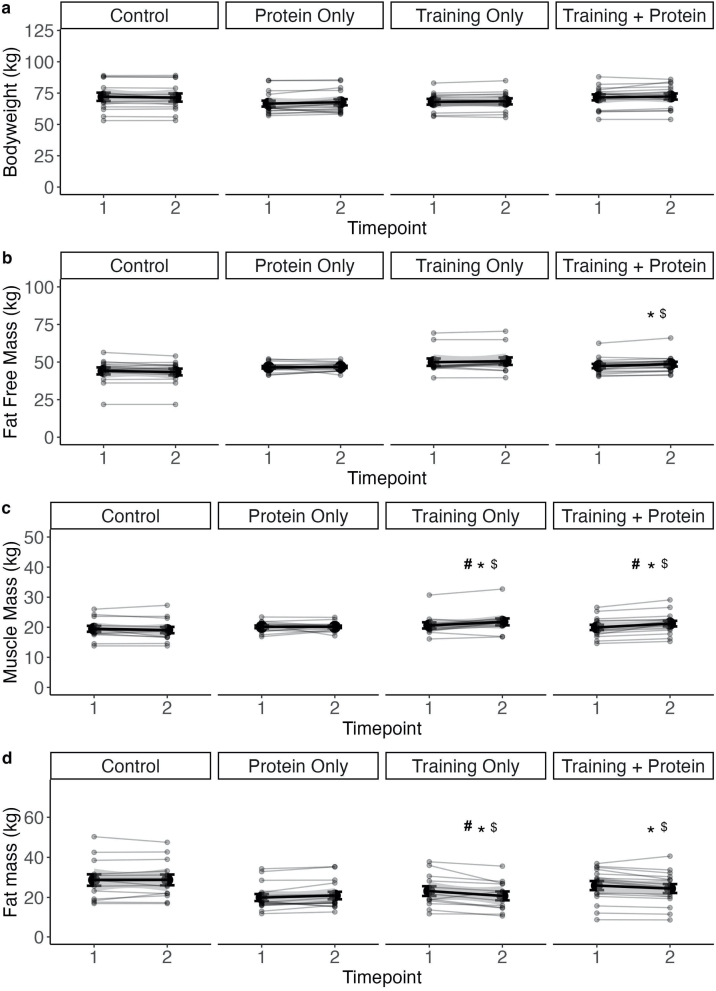
Change in bodyweight and body composition. TP = Training + Protein T = Training, CP = Control + Protein, C = control. Significant time and time*group effects were set p ≤ 0.05. Time effects were marked with # and time*group effects to C with * and to CP with $. Mean values with standard deviation are marked in black and individual curves in grey.

The bodyweight of all four groups did not change significantly over time (p > 0.05) and there was no significant time*group interaction (p > 0.05) (Fig. 2a).

#### 3.2.2 Fat-free mass (FFM)

No significant change over time was observed in the FFM. A time*group difference was found between TP to CP (p < 0.05) and C (p < 0.05). No further time*group differences could be determined (Fig. 2b).

#### 3.2.3 Skeletal muscle mass (SMM)

TP and T increased significantly SMM over time (p < 0.05). For CP no significant effect was detected (p > 0.05). In C no significant change was observed (p > 0.05). Pairwise comparisons of the Δ′s showed a significant difference between TP to CP (p < 0.05) and to C (p < 0.05). Also, T has a significant time*group effect to CP (p < 0.05) and C *(*p < 0.05). No significant difference was observed between TP and T (p > 0.05) (Fig. 2c).

#### 3.2.4 Fat mass (FM)

The FM decreased only significantly in T over time; (p < 0.05). No significant change over time was observed in any of the other groups. A time*group difference was detected between TP to CP and C (p < 0.05) as well T to CP and C (p < 0.05). No significant difference was observed between TP and T (p > 0.05) (Fig. 2d).

### 3.3 Muscle thickness

#### 3.3.1 M. rectus femoris

**Fig. 3:**
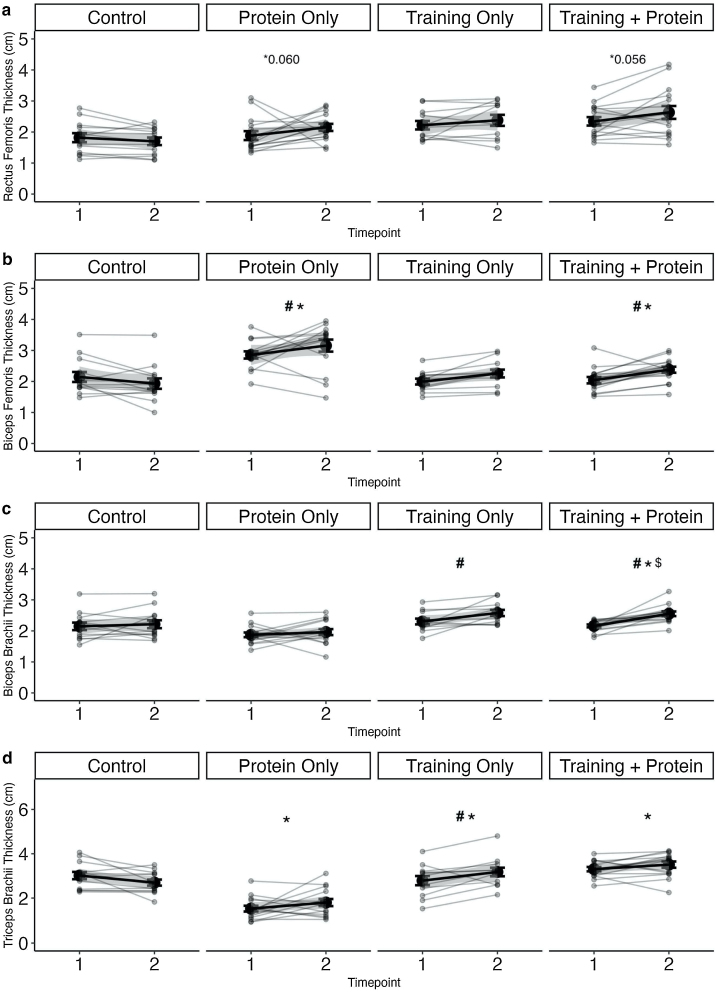
Muscle thickness of M. rectus femoris, M. biceps brachii, M. biceps femoris, M. triceps femoris. TP = Training + Protein T = Training, CP = Control + Protein, C = control. Significant time and time*group effects were set p ≤ 0.05. Time effects were marked with # and time*group effects to C with * and CP with $. Mean values with standard deviation are marked in black and individual curves in grey.

There was no significant time effect for the M. rectus femoris in any group (p > 0.05). Also, no significant time*group interaction was observed (p > 0.05) but two trends were detected between C and TP (p = 0.056) and CP (p = 0.060). No significant difference was observed between TP and T (p > 0.05) (Fig. 3a).

#### 3.3.2 M. biceps femoris

TP and CP significantly increased over time (p < 0.05). T and C showed no significant change over time (p *> 0.05*). Pairwise comparisons of the Δ′showed significant differences between TP to C (p < 0.05) and CP to C (p < 0.05). No significant difference was observed between TP and T (p > 0.05) (Fig. 3b).

#### 3.3.3 M. biceps brachii

A significant increase in M. biceps brachii was observed in both training groups over time (p < 0.05). CP and C showed no significant changes over time (p > 0.05). Comparisons of the Δ′s showed significant differences between TP to C (p < 0.05) and TP to CP (p < 0.05). No significant difference was observed between TP and T (p > 0.05) (Fig. 3c).

#### 3.3.4 M. triceps brachii

A significant time difference could only be determined in T (p < 0.05). All other groups showed no significant change over time (p > 0.05). A time*group difference could be observed between TP to C (p < 0.05), T to C and CP to C (p < 0.05). No significant difference was observed between TP and T (p > 0.05) (Fig. 3d).

### 3.4 Static strength

#### 3.4.1 Grip strength

**Fig. 4:**
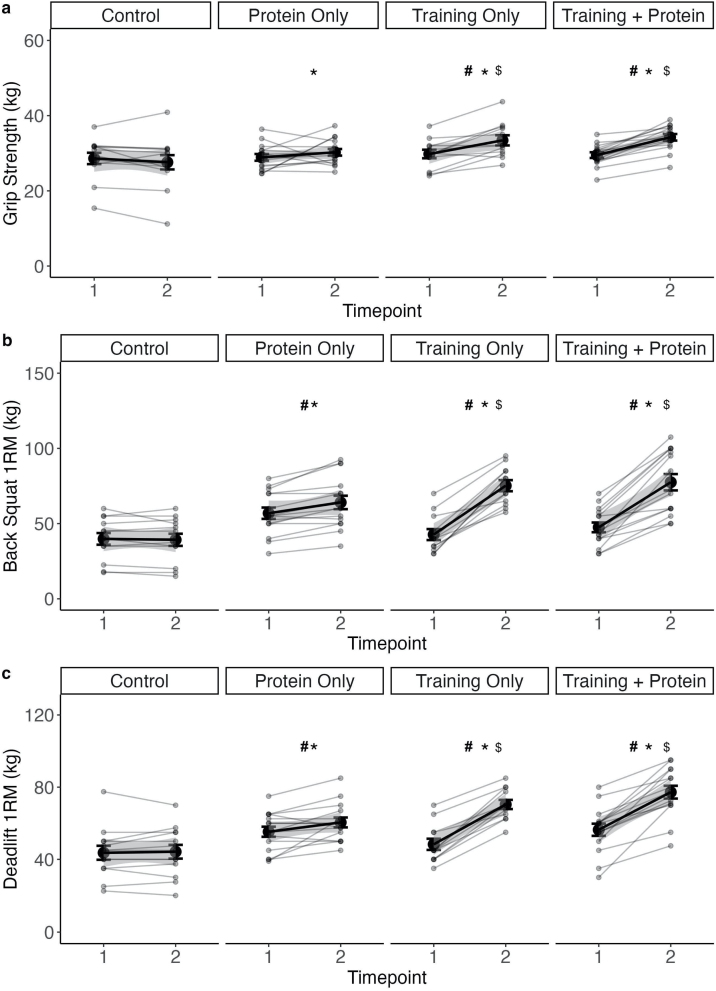
Changes in performance, Grip strength, 1-RM back squat and 1-RM DL GS. TP = Training + Protein T = Training, CP = Control + Protein, C = control. Significant time and time*group effects were set p ≤ 0.05. Time effects were marked with * and time*group interaction to C with * and CP with $. Mean value with standard deviation are marked in black and individual curves in grey.

TP and T increased their GS significantly over time p < 0.05). CP and C showed no significant changes over time (p > 0.05). Pairwise comparisons of the Δ′s showed a significant difference from TP to C and CP (p < 0.05). T showed significant changes to C (p < 0.05) and CP to C (p < 0.05). No significant difference was observed between TP and T (p > 0.05) (Fig. 4a).

#### 3.4.2 Dynamic strength

##### 3.4.2.1 1RM BBS

TP, T showed a significant increase in BBS over time (p < 0.05). CP also increased significantly the BBS performance over time (p < 0.05). C showed no significant changes over time (p > 0.05). Pairwise comparisons of the Δ′s showed significant differences between TP to C and CP (p < 0.05), T to C and CP (p < 0.05) and CP to C (p < 0.05). No significant difference was observed between TP and T (p > 0.05) (Fig. 4b).

##### 3.4.2.2 1RM DL

All three intervention groups (TP, T and CP) significantly increased DL performance over time (p < 0.05). C showed no significant changes over time (p > 0.05): Pairwise comparisons of the Δ′s showed significant differences between TP to C and CP (p < 0.05). Group T showed significant differences to C and CP (p < 0.05). CP showed significant differences to C (p < 0.05). No significant difference was observed between TP and T (p > 0.05) (Fig. 4c).

All absolute values and changes for body composition, muscle thickness and performance are shown in Table 2.

**Table 2:** Means ± standard deviations separated by groups, as well as differences (Δ) between PRE and POST, levels of significance (sign.) and effect sizes (ES -Cohens D) of all measurements in body composition (BW, FFM, SMM, FM), muscle thickness (RF, BB, TB, BF), grip strength, 1-RM BBS, 1-RM DL. The significance was set at p < 0.05, time effects are market with #; time*group interaction to C with * and CP with $.

	TP				T				CP				C			
	T0	T1	Δ	D	T0	T1	Δ	D	T0	T1	Δ	D	T0	T1	Δ	D
BW (kg)	71.6 ± 8.9	72.3 ± 9.1	1.3 ± 2.4	---	68.1 ± 7.6	68.4 ± 8.1	0.3 ± 0.9	---	65.3 ± 7.7	66.5 ± 8.2	1.2 ± 2.4	---	73.0 ± 11.5	72.4 ± 11.4	−0.6 ± 1.4	---
FFM (kg)	47.3 ± 5.5	48.6 ± 5.9	1.3 ± 1.6*	---	49.9 ± 8.1	50.6 ± 8.3	0.6 ± 2.6	---	46.2 ± 3.3	46.5 ± 2.8	0.3 ± 2.2	---	44.6 ± 7.9	43.8 ± 7.6	−0.8 ± 1.5	---
SMM (kg)	**19.8 ± 3.3**	**21.2 ± 3.5#**	1.4 ± 0.9*$	**0.4**	**20.6 ± 3.4**	**21.8 ± 3.8#**	1.2 ± 1.3*$	**0.3**	20.1 ± 1.7	20.1 ± 1.5	0.0 ± 1.5	---	19.6 ± 3.3	19.2 ± 3.5	−0.4 ± 1.0	---
FM (kg)	25.9 ± 8.5	24.4 ± 8.4	−1.5 ± 2.0*$	---	**23.2 ± 8.1**	**20.8 ± 7.4#**	−2.4 ± 2.9*$	**0.3**	18.9 ± 5.7	19.9 ± 6.0	0.9 ± 1.8	---	29.1 ± 9.9	29.2 ± 9.4	0.2 ± 1.7	---
RF (cm)	2.3 ± 0.5	2.6 ± 0.8	0.3 ± 0.6	---	2.2 ± 0.5	2.4 ± 0.4	0.2 ± 0.4	---	1.9 ± 0.5	2.1 ± 0.4	0.3 ± 0.7	---	1.9 ± 0.5	1.7 ± 0.4	−0.1 ± 0.2	---
BF (cm)	**2.0 ± 0.4**	**2.3 ± 0.4#**	0.3 ± 0.4*	**0.9**	2.0 ± 0.3	2.3 ± 0.4	0.3 ± 0.2	---	**2.8 ± 0.4**	**3.1 ± 0.8#**	0.3 ± 0.8*	**0.5**	2.2 ± 0.7	2.1 ± 0.7	−0.1 ± 0.5	---
BB (cm)	**2.2 ± 0.2**	**2.6 ± 0.3#**	0.4 ± 0.3*$	**1.6**	**2.3 ± 0.3**	**2.6 ± 0.4#**	0.3 ± 0.3	**0.9**	1.8 ± 0.2	1.9 ± 0.3*#	0.1 ± 0.4	---	2.2 ± 0.4	2.2 ± 0.4	−0.1 ± 0.4	---
TB (cm)	3.3 ± 0.4	3.5 ± 0.5	0.2 ± 0.4*	---	**2.8 ± 0.7**	**3.2 ± 0.6#**	0.5 ± 0.3*	**0.5**	1.4 ± 0.4	1.7 ± 0.6*	0.4 ± 0.7*	---	3.0 ± 0.6	2.7 ± 0.5	−0.3 ± 0.5	---
GS (kg)	**29.5 ± 2.8**	**34.2 ± 3.3**	4.7 ± 2.4*$	**1.5**	**29.8 ± 3.8**	**33.4 ± 4.5#**	3.6 ± 3.0*$	**0.8**	28.4 ± 2.8	30.0 ± 3.3	1.7 ± 3.9*	---	29.2 ± 5.4	28.0 ± 6.5	−1.1 ± 2.2	---
1-RM BBS (kg)	**47.5 ± 12.1**	**77.5 ± 20.3#**	30.0 ± 14.2*$	**1.5**	**42.7 ± 12.1**	76.7 ± 12.1*	34.0 ± 12.0*$	**2.4**	**57.2 ± 14.5**	**64.7 ± 17.1#**	7.5 ± 5.4*	**0.4**	40.8 ± 16.5	40.4 ± 14.1	−0.4 ± 3.5	---
1-RM DL (kg)	**56.4 ± 13.0**	**77.2 ± 13.3#**	20.8 ± 10.3*$	**1.6**	**48.3 ± 10.3**	70.4 ± 8.4*	22.1 ± 7.6*$	**2.0**	**54.9 ± 10.7**	60.5 ± 10.96*	**5.5 ± 7.7**	**0.5**	44.8 ± 13.6	45.4 ± 13.4	0.5 ± 3.9	---

C = control; CP = Control + Protein. D = Cohens’ D; T = Training; TP = Training + Protein; Significant time and time*group effects were set p < 0.05. Time effects were marked with # and time*group effects to C with * and CP with $.

### 3.5 Liver and kidney function

A significant increase in ALT concentration was observed in CP after 12-week intervention (12.7 ± 5.1 U/L–19.5 ± 7.3 U/L p < 0.05; *d* = 1.2). No significant changes were observed in AST (28.7 ± 15.5–26.0 ± 7.2 U/L; p > 0.05) and GGT concentrations (18.6 ± 6.4 U/L–21.7 ± 12.1 U/L; p > 0.05). There was also no significant difference in the creatinine concentration (0.9 ± 0.1–0.9 ± 0.1 mg/dl; p > 0.05).

### 3.6 Nutrition strategies

**Fig. 5:**
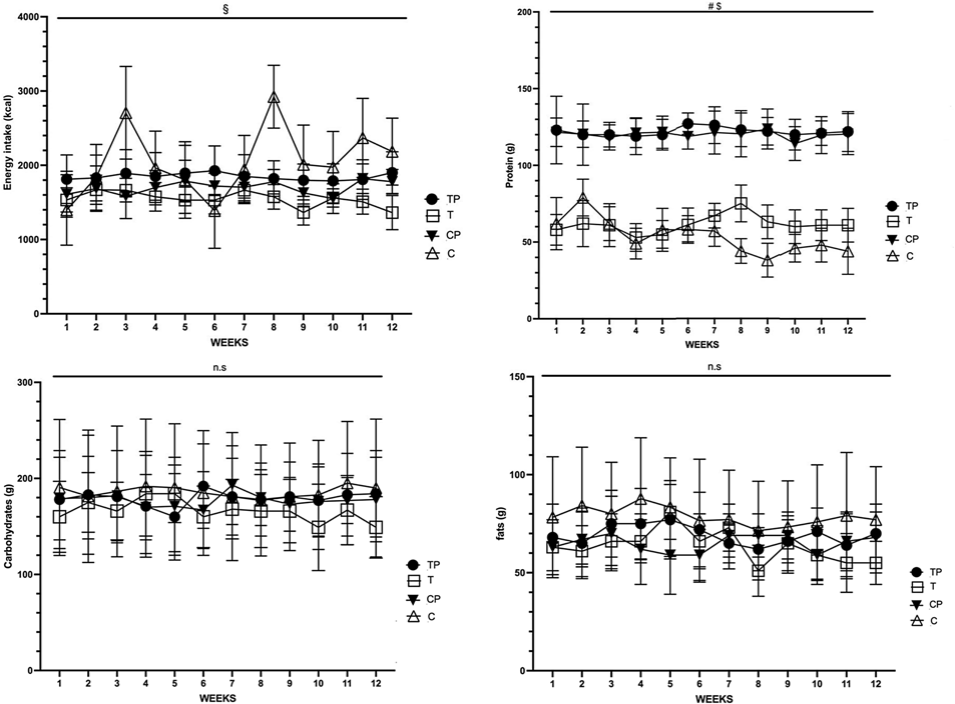
Energy intake, Protein intake, carbohydrates and fat intake over 12 weeks, TP = Training + Protein T = Training C = control, CP = Control + Protein. Significant differences were set p < 0.05, group effect to C market with # and T with §.

The self-documented calorie intake, protein, carbohydrate and fat content of the diet were analyzed. If information on the participants’ nutritional status was lacking for specific days (fewer than eight days in total over 12 weeks), the data from the previous day was used for the evaluation. The TP consumed an average of 1852.4 ± 370.7 kcal per day. T consumed an average of 1708 ± 357.0 kcal per day. The CP consumed an average of 1965.3 ± 578.8 kcal and the C group had an average of 1703.8 ± 244.5 kcal. Both protein supplementing groups showed a significant group difference (p < 0.05) for calorie intake and a highly significant difference in their absolute protein intake (p < 0.05) to the non-supplementing groups T and C. The protein consumption in both protein supplementing groups showed a consequent execution of a high protein diet of TP consuming an average of 121.9 ± 22.4 g over 12 weeks and CP consuming an average of 119.8 ± 15.7 g. The protein intake of the T group was on average of 62.5 ± 17.0 g and for C 53.8 ± 15.6 g. The absolute and relative protein intake to BW and FFM are shown in Table 3. The mean carbohydrate intake of TP was 179.6 ± 58.1 g, for T was 208.1 ± 59.3 g, for CP was 185.4 ± 80.2 g and for C was196.3 ± 45.0 g. The mean fat intake of TP was 67.4 ± 21.6 g, T consumed on average 69.0 ± 19.4 g, CP 79.4 ± 36.6 g and C 74.9 ± 18.7 g (Fig. 5).

**Table 3:** Protein Intake of all Groups.

Group	TP	T	CP	C
Protein (g)	121.9 ± 22.4	62.5 ± 17.0	119.8 ± 15.7	53.8 ± 15.6
Rel. Protein (g/kg BW)	1.7 ± 0.0	0.9 ± 0.1	1.8 ± 0.0	0.7 ± 0.1
Rel. Protein (g/kg FFM)	2.6 ± 0.1	1.3 ± 0.1	2.6 ± 0.0	1.2 ± 0.2

## 4 Discussion

This study aimed to investigate the influence of RT with free weights and the influence of HPD on body composition, muscle thickness and strength capacity. In addition, initial results of HPD on liver and kidney function in postmenopausal women should be obtained. It has been shown that RT with free weights is a safe method with moderate to strong effects on strength capacity and trivial to moderate effects on body composition and muscle thickness. HPD alone had also a small effect on strength capacity.

Maintaining the SMM is important for prolonging functionality and strength in older age [56]. Exercises like everyday movements and maintaining or promoting strength are ideal strategies. This study demonstrates that RT with free weights is a safe method to increase strength and SMM in postmenopausal women. As in previous studies, no high dropout rate could be attributed to the training method [9]. This study was the first to show that RT with free weights significantly increases SMM while reducing FM in postmenopausal women. A significant increase in SMM was observed in both training groups but not in the CP group. An increase of more than one kilo was even observed in both groups. Besides a time*group effect could also observed for TP in FFM to C. These effects are similar to those seen in younger women [57]. Interestingly, the results suggest that postmenopausal women need at least three training sessions per week to increase FFM and SMM. In prior studies with two sessions per week with a similar study design, no significant effect was achieved in FFM and SMM in postmenopausal women but in premenopausal women [9]. However, the significant increase in SMM can be confirmed by the results of Rossato et al. [16]. The TP and CP groups from this study consumed significantly more protein than both groups of Rossato et al. The high protein group von Rossato et al. consumed only 1.2 g/kg BW [16]. Even if a higher absolute value was achieved in the FMM and SMM in the TP than in T, no significant difference between the groups was detected. Consequently, regular strength training is probably the decisive factor for maintaining or increasing FMM or SMM in untrained postmenopausal women. HPD presumably plays a further role only after a longer period than 12 weeks or at a higher performance level. The increase in SMM is even in line with those of elite athletes [57,58]. Consequently, hypertrophy effects can be also achieved with a similar effect to those of young athletes in postmenopausal women with RT. This leads to speculation that female sex hormones are not the exclusive factors for hypertrophy in women. It is known that other hormones such as IGF-1 or insulin, and specific amino acids like l-leucine, can activate the crucial signaling cascade via mTORC1 and consequently promote cell proliferation in muscle cells [[59], [60], [61], [62]]. Accordingly, it can be assumed that these signaling cascades are not significantly influenced by the menopausal transition in women, unlike the concentrations of sex hormones such as estradiol and progesterone.

In contrast to previous studies, a reduction in FM was also observed in both training groups. Even though three training sessions per week were also carried out in the study by Rossato et al., no reduction in FM was observed. One potential reason for this could be the different selection of exercises and intensities [16]. In comparison, this study mainly used moderate to very high intensities (>70%), which presumably created a different stimulus. However, the results also indicate that at least three sessions per week must be carried out to reduce FM. However, in our study it was observed that an HPD increased the total calorie intake in both HPD groups and consequently the TP group lost less FM than the T group. It is therefore crucial that with an HPD, the additive amount of protein is not consumed in excess, but that the calorie balance remains the same by reducing carbohydrates or fats. Interestingly, similar to the C group, no significant changes in CP were observed compared to the training groups. This underlines that RT particularly improves body composition and not a HPD without any other targets such as caloric restriction. In addition to the increase in FFM and SMM, a significant increase in muscle thickness was observed. In the lower body, a significant increase was observed in the TP and CP with a trivial to small effect in the BF. A similar trend was observed for both groups in the RF. In contrast, no significant change was observed in either muscle in T. Although no significant change was observed in T, the individual and average changes indicate that the muscle cross-section was also increased. Besides no significant differences were observed between TP and CP compared to T. However, the results suggest that in addition to intensive RT, protein intake also plays an important role for muscle thickness in postmenopausal women. It is remarkable that similar effects in the lower body muscle thickness as in young, trained men could be achieved [24,63]. This is consistent with previous studies that both men and women can achieve similar adaptations [64,65]. In the upper body, small to moderate significant increases were observed in the BB in both training groups without group differences. In contrast, no clear effects were observed in the TP. This could be due to the fact that the training protocol did not include explicit elbow extension exercises and the potential effects can be attributed to global adaptations. Although the muscle thickness measurement is a valid method for hypertrophy [29,66], measurement inaccuracies could also have led to different effects. In comparison to the training groups, however, CP and C did not show any significant changes over time in the upper body. Finally, the results of the sonography support the significant changes of both training groups in the results of the bioelectrical impedance analysis. Similar to FFM and SMM, the results indicate that HPD has a further influence on muscle thickness, but this cannot be statistically verified. Consequently, the decisive factor for muscle thickness is also regular intensive RT in women who are not experienced in resistance training.

In addition, a significant increase in strength capacity was observed. Both training groups showed a significant improvement in the dynamometer, whereas no significant changes were observed in the CP and C groups. Grip strength is an important indicator of muscle strength and muscle functionality [[67], [68], [69], [70]]. However, it has been observed in previous studies that a strong adaptation can only be achieved through repeated measurements [9]. Although a familiarization took place at the beginning of the study, potential adjustments due to the measurement repetition cannot be excluded. In contrast, the dynamic strength of the BBS and DL showed moderate to strong significant improvements in the TP and T. Although the average absolute increase is almost the same for both training groups, different effects were observed. This could be due to the different standard deviations and the individual increases. In addition, no significant difference was found between the two groups.

Like previous studies, the maximum strength in the lower body was increased by more than 20 kg [9,16]. However, although a higher training frequency and volume were performed to previous studies, no clear stronger effects were observed compared to two training sessions per week [9]. Besides the increases in dynamic maximum strength parameter were similar to those seen with young men and women [24,64,71]. Interestingly, a significant increase in BBS and DL was also observed in the CP group. This can be supported by previous studies [13,15,72]. Reasons for the potential adaptation could be due to the continuous positive protein net balance, so that muscle quality is maintained for longer. Although there are initial studies and guidelines for checking muscle quality using ultrasound measurements [73], there is currently a lack of further studies and measurements to verify this. However, it appears that HPD without training also has positive effects on strength, but no additive effects could be observed in combination with RT.

In addition to the main parameters of body composition and strength, the CP group was also monitored for liver and kidney function. No pathological changes in liver and kidney function were detected. This is consistent with previous studies of super HPD over one year in young healthy men [19]. However, a significant increase was observed in the ALT concentration after 12 weeks of HPD. Even if there is no pathological change, it could be shown that there is higher liver activity. It is known that liver and kidney functionality can decrease with the ageing process [[20], [21], [22], [23]] and that this could possibly lead to potential side effects due to an increased protein intake. However, these speculations need to be confirmed in future studies with older people. At present, however, it is still recommended to consume a sufficient protein intake of 1.0–1.2 g/kg BW to prevent sarcopenia [74]. Even if slight differences between TP and T can be recognized in individual parameters, these cannot be confirmed statistically. This means that HPT currently has no additive effect in postmenopausal women with no experience in RT over a period of 12 weeks. The effects of HPD on body composition, muscle thickness and strength in strength-experienced postmenopausal women or over a longer period of time, as well as the potential risks to liver and kidney function, must be examined in further studies.

### 4.1 Limitations

In addition to the important and new findings, this study also has some limitations. One aspect is the current lack of standardization of ultrasound measurements in sports medicine contexts. Even though there are already initial position papers on the implementation of ultrasound measurements in clinical examinations [9,29,66,75] these can still be subjectively influenced. Therefore, the same person should perform both the initial and final measurements. In addition, another person should assist with the post-measurement to identify the exact location but should not measure the muscle thickness. Furthermore, the ultrasound results should only be used in correlation with other methods for muscle thickness and hypertrophy to confirm potential effects. Another factor is the self-documentation of the diet. Like other nutritional studies, the actual energy intake and macronutrient distribution cannot be clearly traced. However, since all groups probably have the same measurement inaccuracy, this can be neglected in the overall context. To possibly better standardize the diet, the energy turnover should be determined in the future and food packages should be made available to the participants. Due to resource constraints, this option was not available in the present work. Furthermore, liver and kidney function as well as the hormone profile should continue to be checked before, during and after the intervention to identify possible side effects as quickly as possible.

## 5 Conclusion

This study investigated the effects of RT with free weights and an HPD. The results indicate that RT with free weights is a safe method for postmenopausal women and that strength capacity and SMM could be significantly increased. It was also shown that an HPD has a significant effect on strength capacity without training but no additive effect could be observed with RT. Furthermore, increased liver activity was observed due to an increase in GOT concentration because of HPD. The long-term effects of a HPD in postmenopausal women cannot currently be answered. Further studies on HPD in postmenopausal and older people on liver and kidney activity are therefore necessary to identify potential side effects.

## Competing interests

The authors declare that they have no competing interests.

## Funding

No external funding.
